# The Pleasures of a Water Cure

**Published:** 1854-09

**Authors:** 


*The Pleasures of a Water-Cure.*—Sitting in our office the other evening we were surprised by the entrance of a relative and former patient, who resides some four score miles away. He is suffering from incipient tuberculosis, and, at the earnest entreaties of his wife, had on the morning of his visit, put himself under hydropathic treatment. He gave his experience as follows. H.

"I left the cars at Clifton Springs at 10, in the morning. On arriving at the 'Water Cure,' I found a sick Irishman sitting on the steps, whom I learned to be the porter. He took my trunk up to the fourth story, sitting down occasionally to rest, and lean his head upon his hand. The stairs were covered with rope rugs, with the exception of the last pair, which was painted a cold, dismal blue. My sky-parlor proved to have three other occupants—one, a paralytic; one with a white swelling; and the third with a somewhat offensive carbuncle. Leaving this cheerful scene, I was conducted to the baths, where I had the privilege of witnessing a great variety of cutaneous diseases. All about were poor wretches, hobbling on canes and crutches, and making wry faces over draughts of the brimstone water; all drinking from a single green glass tumbler, the fluid itself tasting like a mixture of rotten eggs and rain water, drank from an old boot, and smelling like a regiment of Scotchmen.

"My next trip was to the doctor, who said I did n't use my lungs below the nipples, which was another cheerful circumstance. The best thing of the day was the dinner, which, by the way, I went up-town to a hotel for, and which was free from the brimstone smell.

"At 2 o'clock, P. M., I ordered my baggage down, and having satisfied the reasonable charges of the doctor, (25 cents, U. S. currency,) I took the cars for Buffalo, having had just four hour's experience of the Water-Cure."

